# Effectiveness of Psychoeducation and Mutual Support Group Program for Family Caregivers of Chinese People with Schizophrenia

**DOI:** 10.2174/1874434600802010028

**Published:** 2008-04-22

**Authors:** Wai Tong Chien

**Affiliations:** The Nethersole School of Nursing, Faculty of Medicine, The Chinese University of Hong Kong, Shatin, Hong Kong SAR, P.R. China

**Keywords:** Psychoeducation, mutual support, group intervention, family caregivers, schizophrenia, Chinese.

## Abstract

Schizophrenia is a disruptive and distressing illness, not only for the person affected but also for family members. Family intervention, particularly in a group format using a diverse range of modalities, is thought to effectively satisfy the informational needs of families and enhance their coping abilities when caring for a relative with schizophrenia, and thus reduce a patient’s relapse from illness. This study tested the hypothesis that participants in a family psychoeducation and mutual support group would demonstrate significant improvements in levels of patient and family functioning and shorter duration of re-hospitalization than families in routine care. A randomized controlled trial was conducted with a sample of 68 Chinese families of schizophrenia sufferers in Hong Kong, who were randomly assigned to either a family psychoeducation and support group (n = 34), or a routine care group (n = 34). The interventions were delivered at two psychiatric outpatient clinics over a nine-month period. Results of multivariate analyses of variance test indicated that the psychoeducation and support group reported greater improvements on family and patient functioning and shorter lengths of patient hospitalizations at the two post-tests (one month and one year after completion of the intervention), compared with the routine care group. The findings substantiate that within a Chinese context, psychoeducation and mutual support group intervention can effectively help families care for a mentally ill relative.

## INTRODUCTION

Schizophrenia is a disruptive and distressing illness, for the people affected and their family members. Similar to the United States [1], over one-third of people with schizophrenia in Hong Kong live with their families, and they often depend on a family member’s assistance and involvement in providing care at home [2]. However, these family members are often inadequately prepared to be the main caregiver for the ill relative [3]. Studies have indicated that there is a severe burden imposed upon the whole family when caring for a member with schizophrenia, because of unpredictable and bizarre behavior, external stressors of stigma and isolation, family conflict, emotional frustration and burnout [4,5].

With the current emphasis on community care for mentally ill patients, family intervention, especially using a diverse range of modalities and a group format, could satisfy the informational needs of families [6], and develop a variety of coping strategies ensuring effective care is provided for a relative with schizophrenia [7], and thus patient relapses are ultimately reduced [8]. Although there have been a few theoretical and psychological models of commonly used family group interventions, empirical studies seeking to explain which model is most effective have been inconsistent. Two recent systematic reviews of family interventions in schizophrenia suggest that a few psychological models [8,9], such as psychoeducation family groups and behavioral family management, reduce patient relapse and readmission but not family distress and burden. In addition, most family intervention studies have focused on Caucasian populations; few have included Hispanics and Asians [10,11]. There are also only a limited number of studies focusing on Chinese populations, even though they may be more likely to be affected by their interactions with family members [12]. Therefore, it is unclear whether interventions that have been established as effective in Western countries can be applied successfully to a Chinese family culture. This study sets out to systematically find an effective model of intervention for Chinese families caring for a mentally ill member.

## LITERATURE REVIEW

### Models of Family Intervention in Schizophrenia

Research over the last 20 years has established a sound evidence base for the effects of the family environment on mental illness [13]. Based on psychological and sociological theories, family dysfunction and over-emotional and critical environments clearly contribute to the emergence of schizophrenia, affect its course and influence the achievement and maintenance of treatment gains by patients [14]. Family intervention for individuals with schizophrenia, which was originally built on research on expressed emotion, has thus received much attention by mental health practitioners and researchers worldwide [2,15]

Because of changes in the organization of mental health services in both Western and Asian countries [4,16], the past decade has witnessed a substantial increase in demands for family interventions within the community as well as a rapid growth of a variety of family intervention models, which have been influenced by behavioral and cognitive psychological therapies [13]. All family intervention programs offer psycho-education and psychosocial support to family members, and some include the patient, although the theoretical orientation of these interventions varies considerably. Common elements in psycho-educational group interventions include the involvement of the family in: (i) patient care provision, (ii) presentation of information about the mental illness and its management, (iii) discussion of the techniques for patient care such as communication, problem solving, medication compliance, and crisis intervention, and (iv) development of social network and coping skills [10]. Moreover, intervention studies have produced inconsistent or inconclusive effects on patients, other than in terms of delaying relapse and improving drug compliance [8]. Surprisingly, few clinical trials of family intervention have assessed family-related outcomes [9], and those that had have reported inconsistent findings about any significant improvement in family functioning.

A few studies have compared different types of approaches to family intervention, but they do not indicate which particular treatment model or combination of techniques from different models is the most effective in helping family caregivers cope better with their caring role and enabling them to have a satisfactory family life [10,17-19]. Barbato and D’Avanzo [9] suggest that the widely held belief that effective family intervention consists of a clearly defined set of psycho-educational and/or cognitive-behavioral techniques following a step-by-step skill building format, is unwarranted. However, the multiple family group approach and patient involvement in the group intervention are evidenced to contribute to significant positive outcomes such as reducing patient relapse and re-hospitalization and improving family relationship [8,10]. In fact, the benefits of these approaches and components of family intervention for family members and their relatives with schizophrenia are not conclusive [11].

Anderson and Adams [20] suggest that the hesitation of clinicians to use family interventions may be related to concerns about the experience and training required to become a therapist. Even though some potential effects of family intervention can be found in routine practice, patients and families may not be willing to spend sufficient time in contact with the intervention to gain what may be seen as modest benefit [8]. There is room for further development and evaluation of an effective pragmatic family intervention program for schizophrenia that can produce more valid and consistent effects on patient and family functioning over a reasonably long period [9].

### Educational & Support Groups for Family Caregivers

The proliferation of family educational and supportive groups in the United States and other Western countries in the 1990s was part of the larger social movement of self-help agencies or organizations for people affected by a variety of chronic diseases and stressful life circumstances such as diabetic, developmentally impaired and alcoholic patients, whose needs had been inadequately addressed by traditional health care interventions [1]. Mutual support is a participatory process of sharing common situations, problems and experiential knowledge about common concerns within a group session [21].. Mutual support groups are characterized as client-led social or community alternatives to the professional-controlled medical programs that dominate the mental health system today. Increasing research evidence indicates that participation in mutual support groups for patients with chronic physical or mental illness, and/or their families, is highly associated with general improvements for patients and psychological adjustment of their families [22-24]. This growth of mutual support groups had led to increased interest in extending their use and examining their value for family care of people with schizophrenia and other severe mental illnesses [22].

Social support theory holds that social support and social network may promote mental health through two major mechanisms. Social support may: (a) buffer the effects of stressful life events; and (b) directly influence the occurrence of various mental disorders [25]. Cohen and Wills [25] reviewed 13 family studies of mentally ill people and concluded that emotional and instrumental support from intimate social interactions can have a potential buffering effect on the stressful events that are associated with caregiving. Family caregivers may be willing but ill-prepared for the physical, emotional and financial burden on family resources due to the illness of a relative. Mutual support groups often facilitate on-going information dissemination through mutual sharing amongst group members [26] thus empowering family members with knowledge and ability to manage the illness, and enhance their chance of living a life that is as normal as possible.

Psychoeducation group programs for families who care for a relative with schizophrenia or other severe mental illness have been commonly used and found effective in research among Western and Chinese populations [4,27]. Chien and Wong [7], when describing the successful psychoeducation program used in their study, highlighted the importance of a family needs assessment, the encouragement of peer support between participants, and adequate staff training as well as on-going supervision. Gidron *et al*. [28] indicated that educational and supportive group participation of families who are caring for a relative with severe mental illness enhances the knowledge of the illness, its treatment and mental health services as needed, and encourages the utilization of the social resources available and the employment of more frequently active and interactive help-seeking coping strategies. However, Brady *et al*. [29] found no significant differences reported by family support group participants over non-participants in terms of satisfaction with personal adaptation to the illness and improvement of the family environment. Norton *et al*. [30], on the other hand, found that family support groups provided guidance on how to establish better relationships with relatives with severe and enduring mental illness and how to negotiate more effectively with the mental health system. Hence, it remains inconclusive whether involvement in a psychoeducation and mutual aid group is associated with positive health-related outcomes such as reducing caregiving burden and improving family interactions and relationships for families who care for schizophrenic patients.

Mutual support and education groups for families of people with schizophrenia have been subjected to many qualitative and quasi-experimental studies, which confirmed the apparent benefits of maintaining the psychological and social well being of families [1,16]. There is relatively little empirical evidence that supports the enthusiastic claims for their benefits in improving family functioning and satisfying families’ psychosocial needs [31]. In Chinese communities, psycho-educational and behavioral management programs are the most commonly used family intervention model for patients with schizophrenia [32,33], though few studies have used mutual support groups as a major component of the psychosocial intervention. In treating Chinese families, it is important to recognize, respect and utilize the culture-specific family structures, functions and processes, such as the extended family structure with close linkage and interrelationships, interdependence and a strong sense of filial responsibility, collective identity and tangible support [15]. There is a need to adapt the family intervention that has originated in the West to take into account Chinese ways of communication (as characterized by an emphasis on mutual respect and positive action for family members rather than talking) and to establish a therapeutic relationship between group members.

### Study Rationale

A few studies of family psycho-education have demonstrated lower relapse rates in people with schizophrenia from Asian backgrounds than from Anglo-Saxon or Afro-Caribbean backgrounds [12,34]. Culturally sensitive, pragmatic trials of psychoeducation and support groups with mutual aid and helping concept must be undertaken to settle arguments about the value of family intervention in Chinese patients with schizophrenia. This form of family intervention has a preliminary evidence base, with several exploratory and descriptive studies focusing on families of patients with schizophrenia and other severe mental illness, and such intervention, when compared with other treatment models such as cognitive and behavioral therapies, requires relatively less intensive training of psychiatric nurses or other health professionals as facilitators,. Educational and supportive groups also provide a flexible, participant-directed approach to helping family caregivers cope with their caregiving role, develop their competence in handling caregiving situations, increase their knowledge of the illness, and improve the consequences of caregiving in family life [9]. It is therefore important to test the efficacy of a psychoeducation and mutual support group program for Chinese families caring for a relative with schizophrenia.

This study sought to test and compare the effectiveness between two modes of community-based interventions, namely: (i) a family psychoeducation and mutual support group and (ii) the conventional psychiatric outpatient care (routine care group) for family caregivers of people with schizophrenia in a Chinese population. The two groups were compared prior to (Time 1), one month later (Time 2) and 12 months after the intervention (Time 3), and multiple outcome measures for families and patients were used to assess treatment effects. The main hypothesis was that the psychoeducation and support group intervention would significantly reduce schizophrenic patients’ psychosocial functioning and lengths of re-hospitalizations as well as improve families’ caregiving burden, functioning, perceived social support, and service utilization, when compared with the routine psychiatric care in Hong Kong at 12-month follow-up.

## METHODS

A repeated measures design was used to compare two groups of family caregivers of patients with schizophrenia: a psycho-education and support group and a standard care (conventional psychiatric outpatient care) group. This study was undertaken between January 2005 and September 2006. Analysis of data was on an intention-to-treat basis [35]. All subjects, who completed the intervention or not, were followed up over a 12-month post-intervention period. They were selected randomly from two regional outpatient clinics in Hong Kong, consisting of about 1,800 patients with schizophrenia or its subtypes, representing 12% of this type of patients in Hong Kong [36].

### Sample & Study Settings

Sixty-eight of 300 eligible subjects were selected randomly from a list of family members who were the primary carers of a relative with schizophrenia attending one of two major psychiatric outpatient clinics in the New Territories (comprising 8% of the total patient population) in Hong Kong. Based on previous studies on family psycho-education group in Chinese population [2,7], a sample of 34 participants in each group was required to detect any significant difference between the groups at a 5% significance level with a power of 80% [37], taking into account potentially 20% of attrition. All the families that met the following inclusion criteria were invited to participate:

- Families living with and caring for one relative with a primary diagnosis of schizophrenia, according to criteria of the Diagnostic and Statistical Manual of Mental Disorders, 4^th^ edition, DSM-IV [38];
- The relative with schizophrenia did not suffer comorbidity of other mental illness during recruitment to the study and who had been diagnosed with schizophrenia for three years or less; and
- Those were aged 18 years or over and able to understand and read the Chinese language.

Exclusion criteria included those who cared for more than one family member with mental illness, who themselves had mental illness, and who were the primary carers for less than three months. Those eligible were listed in alphabetical order by surname. They were then selected randomly from the patient list, using a computer-generated random numbers table.

For those patients with more than one caregiver, the research group recruited the family member identified him/herself as the primary carer with the most important caring role. Of these, 64 families signed an informed written consent and voluntarily participated in the study. They were then randomly assigned to one of the two groups in this study: psychoeducation and mutual support group (n = 34) or standard care (n = 34).

### Data Collection

**Fig. (1):**
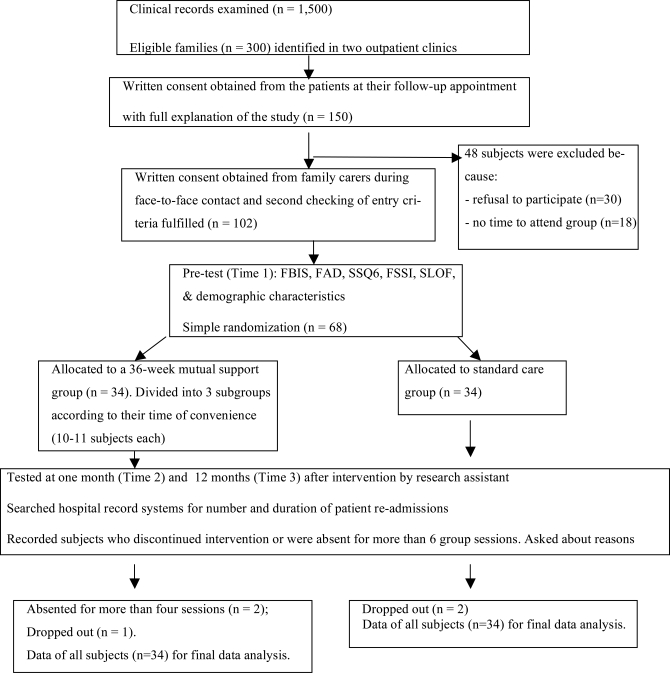
Flow Diagram of clinical trial for family psychoeducation group & standard care.

Enrolment, allocation, treatments, measures, and analyses of data from the study subjects are summarized in a flow diagram (Fig. **1**), according to the revised version of CONSORT statements [39]. A trained psychiatric nurse (group instructor) approached the patients in person during follow-up appointment to seek written consent for voluntary participation in this study and permission to approach their family members. With the patient’s permission, one family member who was the primary caregiver of the patient was contacted by telephone to explain the purpose and procedure of the study and to invite his/her participation in the study. Written consent was obtained in a face-to-face interview and the participants were then asked by the principal researcher to draw a sealed opaque envelope, in which a number card indicated to which group they had been allocated (1 = psycho-education group and 2 = routine care group). Except for the principal researcher and the group instructor, all other clinic staffs were blinded to treatment allocation. An independent assessor (a research assistant) was trained to undertake measurements at Time 1, 2 and 3 using a set of questionnaires.

### Instruments and Outcome Measures

Subjects completed a battery of the Chinese versions of four scales: Family Burden Interview Schedule (FBIS) [40], Family Assessment Device (FAD) [41], Family Support Services Index (FSSI) [42], and Specific Level of Functioning Scale (SLOF) [43]. Demographic data were also collected. The questionnaires took about 45 minutes to complete.

The FBIS [40] is a 25-item semi-structured interview schedule to assess the burden of care placed on families of a psychiatric patient living in the community. It consists of six categories of perceived burden (2-6 items in each category), including effects on family finance, routine, leisure, interaction, physical health and mental health. The items are rated on a 3-point Likert-type scale (‘0’- ‘No burden’, ‘1’ - Moderate burden’ and ‘2’ - ‘Severe burden’). The total scores range from 0 to 50, with higher scores indicating higher burden of care. Satisfactory inter-rater reliability and significant correlations with both clinical psychopathology and social dysfunction in the patient have been shown [44]. It was translated into Chinese language and validated by the researchers with 185 family members of patients with schizophrenia in a pilot study. This Chinese version demonstrated strong internal consistency for total score (α = .87), and high inter-rater coefficients (.87 to .99 for items and .72 for total score).

The FAD [41] was used to assess multiple dimensions of family functioning among patients with mental disorders and other unhealthy conditions and was based on a well-developed theoretical and family treatment model. It consists of 60 items to measure family functioning on a 4-point Likert-type scale (1- ‘strongly disagree’, and 4- ‘strongly agree’) along seven dimensions: problem solving, communication, roles, affective responsiveness, affective involvement, behavioral control, and general functioning. A Chinese version of the FAD [45] used in this study indicated high content validity when reviewed by experts and we had satisfactory internal consistencies for families of patients with schizophrenia in Hong Kong (.68 to .92 for dimensions and .97 for overall scale). The possible total scores range from 4 to 28, a higher score reflecting poorer functioning of the family.

The FSSI [42] is a checklist to measure needs and usage of formal support services by psychiatric patients and their families. It was translated into Chinese language according to the available family support services for psychiatric outpatients in Hong Kong, by checking the service list obtained from the community psychiatric nursing team. An expert panel of psychiatrists, community psychiatric nurses and medical social workers reviewed and attested the appropriateness of the list and its translation to Hong Kong setting, except for one item (in-home respite service), which was deleted. The modified index contained 16 items concerning family support services and each item was rated for whether the family was in need of it (Yes/No) and whether they were receiving it (Yes/No). Inter-rater and internal reliabilities were .88 and .84, respectively [2]. The responses to this scale indicate the number and types of services that families are in need of and receiving.

The SLOF [43] is a 43-item assessment scale, which comprises three main functional areas for patients with schizophrenia: self-maintenance (12 items), social functioning (14 items) and community living skills (17 items). A Chinese version [46] has displayed satisfactory content validity, test-retest reliability (r = .76), and internal consistency (α = .88 to .96 for its subscales) for Hong Kong Chinese patients with schizophrenia or other psychotic disorder.

All families also completed a demographic data sheet, which included their age, gender, educational level, biological relationship with patient, monthly household income, and number of family members living with patient; as well as the patient’s age, gender, duration of mental illness, present medication, and mental condition (improved, stable/staying the same, or worsened/not stable) in the last three months.

The number and duration of psychiatric hospital admissions, during the preceding six months at the start of the group intervention (Time 1), over the nine-month period of intervention (Time 2), and over the 12-month period after intervention (Time 3), which were obtained from the hospital and outpatient clinic record systems.

### Psychoeducation & Mutual Support Group

Participants (n = 34) received a 36-week program of mutual support and the conventional psychiatric outpatient care. The group met on a bi-weekly basis for 18 sessions (over nine months), each lasting about two hours (Table **1**).

The psychoeducation component of the program was similar to that described by Posner *et al*. [47] and the mutual support component was outlined according to the recommendations by Galinsky and Schopler [48] and Wilson [49]. The primary caregiver of the patient recruited as the study participant was asked to attend all the sessions and completed the questionnaires; other family members and patients were invited to attend only parts of the program. Each session began with a didactic presentation using Microsoft PowerPoint (for Windows) presentation or slides, followed by a group discussion that emphasized the significance and relevance of information obtained for their family lives. Caregiving situations and incidents were presented by the caregivers and alternative ways of coping and resolving their difficulties of care provision were discussed among the caregivers. A trained psychiatric nurse with expertise in psychiatric rehabilitation led the three caregiver subgroups, summarized the major findings of their group discussion and provided recommendations on techniques for patient management. Other mental health professionals such as psychiatrists, psychiatric nurse specialists and medical social workers were invited to be guest speakers or co-leaders. Mutual aid and support concepts, accepted by health professionals, were used in five later sessions of this program because this allowed flexibility in time management, task achievement, autonomy, interdependence, and even termination [26,49].

Such family program also met the unique socio-cultural needs of Asian-American schizophrenic patients and their families [12]. Specific Chinese and Asian cultural characteristics were emphasized during each group session. These included the high social stigma associated with mental illness and seeking mental health services, hierarchical but interdependent family structure, preference for indirect communication, difficulties in disclosure of feelings to strangers (at the first few sessions), and high tendency to expect immediate and practical help [31,50].

To work effectively for mutual support in the later sessions, the group instructor continuously reinforced the principles of strengthening social support among the participants [48,49], including: sharing personal data (ensuring confidentiality and disclosing information with trust), dialectical process (let members think about ideas and alternatives to solve problems), discussion of a taboo area (sharing of secret and internal psychological conflicts), commonality or a situation of ‘all-in-the-same boat’ (feeling in similar situation and working against a common plight), mutual help (reciprocal giving and receiving help and support among members), and individual problem solving (helping individual members to address unique troubles). In-meeting and post-meeting rehearsals and practices in caring for their relative with schizophrenia at home was emphasized and evaluated in each of the nine later sessions. Supervision and progress monitoring of this psychoeducation group comprised consistent reviews of the audiotape of each session by the researcher and the group instructor, and regular clarification of problems and issues arising from each group session.

### Routine Care

Participants (n = 34) received the conventional psychiatric outpatient and family services. These services varied very little between the two clinics and included medical consultation and advice, individual nursing support and advice on available community health care services, social welfare and financial services provided by a medical social worker, and counseling by a clinical psychologist if necessary. At completion, as an ethical move, we invited the participants in the routine care group to participate in a similar psychoeducation group should they wish to do so, as the group intervention were effective.

### Data Analysis

Descriptive and inferential statistics were employed on the demographic data and the pre and post-test measurement scores between the two groups, using the Statistical Product and Services Solutions (SPSS) for Windows, version 13.0. Demographic differences between the two study groups were assessed by an independent sample t-test or the z score (i.e., sample size > 25), or? two-tailed, as appropriate.

Between-groups comparison on the baseline scores of dependent variables (FBIS, FAD, FSSI, SLOF, and length of re-hospitalization) at Time 1 using ANOVA was conducted between the two groups. Repeated-measures multivariate analyses of variance test (MANOVA) was performed for the dependent variables to determine whether treatment produced the interactive effects postulated (group x time). Preliminary assumption testing was conducted to check for normality, linearity, univariate and multivariate outliers, homogeneity of variance-covariance matrices, and multicollinearity, with no significant violations noted [51]. Post-hoc analysis using Tukey’s Honestly Significant Difference (HSD) test for multiple comparisons was performed on those measures that indicated a significant interaction effect of time-by-program in the MANOVA. The level of significance for the statistical tests was set at .05.

## RESULTS

### Sample Characteristics

Of the two groups, more than one-half of family caregivers were: females (58.8% and 61.8%), more than two-thirds of them had completed secondary education or above (about 70%) and had monthly household income ranged between HK$10,001 (US$1,282) and 25,000 (US$3,205). Their mean ages in the two groups were 42.1 and 43.2 years (SD = 6.1 and 7.8; age range = 30 – 49 years). Relations with patient were mainly child (29.4% and 32.4%), spouse (both 29.4%) and parent (both 26.5%). Table **2** presents the socio-demographic characteristics of these family caregivers. There was no significant difference on these characteristics among the three groups.

About two-thirds of patients were males (62% - 68%) and had completed secondary school education (59% - 66%). The patients’ mean ages were 37.3 (SD = 6.2) and 28.8 (SD = 7.1) years in the intervention and control groups, respectively (age range = 20 – 49 years). Their mental condition was described as stable (45% - 48%) or worsened (28% - 32%) in the past three months. Over half (51% to 58%) of them were taking medium dosage of anti-psychotics (Haloperidol equivalent mean values between 8.10, SD = 5.6 and 10.1, SD = 8.1), as per the average dosage of medication taken by schizophrenic patients as recommended by the American Psychiatric Association [52]. The average number of family members living with patient was about two (1.8 and 2.3) in the two groups. Mean duration of illness was just 2.5 years (i.e., 7 months to 5. years).

Thirty-one subjects (91.2%) from the mutual psychoeducation and support group completed the program. These subjects, together with those who dropped out or absented in more than four group sessions - psycho-education group (n = 3) and routine care group (n = 2), were evaluated at three times of outcome measurements. Reasons for dropout or discontinuation from the group interventions were mainly: insufficient time to attend (n = 2), patient’s mental state worsened (n = 3), not interested (n = 2), and the only person taking care of patient (n = 2).

### Testing Homogeneity of Groups

As indicated in Table **2**, there were no significant differences on any of the socio-demographic variables between the two groups. Group comparison of the amount and the use of atypical (e.g., clozapine) versus conventional (e.g., haloperidol) antipsychotic medications did not reveal any difference at Time 1, 2 and 3, using ANOVA and Chi-square test, respectively. There were also no significant correlations (r < .15 and p > .10) between the socio-demographic variables and outcome measures, and thus indicated no covariate effect.

### Treatment Effects

Between-groups comparison of the baseline scores of dependent variables at Time 1 using ANOVA test found no significant differences between the two groups. There was a statistically significant difference between the two groups on the combined dependent variables, F (5, 66) = 4.89, p = .005 (Wilks’ Lambda = .90; a large effect with partial eta squared = .48). The results of MANOVA for the dependent variables in the repeated-measures when considered separately (Table **3**) indicated that there were statistically significant differences between groups on: a reduction in the FBIS (burden) score [F (1, 66) = 4.32, p < .01], and an improvement in the FAD (family functioning) score [F (1, 66) = 4.50, p < .01] as well as the patients’ SLOF score [F (2, 95) = 4.68, p < .01]. An inspection of the adjusted mean scores at Time 1 to 3 indicated that psychoeducation group reported continually positive improvements of the FBIS, FAD, FSSI, and SLOF scores, whereas the caregivers in routine care reported minimal changes of scores in the five measures between Time 1 and 3 and a marked deterioration of patient functioning at Time 3. The results of MANOVAs for the subscale scores of the FBIS, FAD and SLOF also indicated that there were statistically significant differences between the two groups in all subscales, except ‘physical health’ in the FBIS. The inter-group mean differences exceeding the minimum significant difference for Tukey’s HSD test procedure were indicated in the following:

- Perceived burden score of the psychoeducation group reduced significantly at Time 2 when compared with the routine care group, while for both groups it remained at a lower level at Time 3 than that at Time 1 and 2;
- Family functioning of the psychoeducation group improved significantly at Time 2 and 3 when compared with routine care; and
- Patients’ level of functioning of the psycho-education group also improved significantly over time from Time 1 to Time 3, when compared with the routine care group. In addition, the SLOF score of the standard care group showed a marked deterioration at Time 2 and remained at a low level at Time 3.

A few of the subjects in the two study groups (i.e., three and four in the psychoeducation and routine care group, respectively) received individual and group therapies provided by the outpatient clinics under study. These programs were weighted in terms of type and frequency of participation and these data were compared between groups to verify whether they differed between the two groups. No significant difference pertaining to participation in other family programs was found, using a two-tailed independent sample t-test.

## DISCUSSION

In this study, the psychoeducation and mutual support group program for family caregivers of Chinese people with schizophrenia demonstrated positive effects at one month and 12 months after the completion of the program on both patients’ and their families’ health outcomes. Further follow-up of the participants will be conducted at 24 and 36 months to see if these effects are sustained. It is interesting and important to note that the attrition rates of the two study groups were very low, when compared with the results in previous studies in Western and Chinese populations [28,30,33]. The high group attendance rate of the family caregivers (and the patients in a few sessions) in both study groups may be explained by the fact that the illness was of short duration and thus the families were likely to be more optimistic and enthusiastic about the potential for positive change during the illness and possible prevention of patients’ relapse [17,18,53]. This also indicates a need for family-centered services to offer accessible and early intervention at home or in the community when patient discharge is being planned to take place in the near future. In spite of such low attrition rate, the participants expressed difficulties in attending the group meetings, mainly due to the inconvenience arising from insufficient time available, and as the main person caring for the patient not having a minder to look after the patient when the carer is participating in the group sessions, particularly when patient’s mental state was unstable.

The results of the psychoeducation program were encouraging and the participants in the program indicated a significant decrease in family burden in terms of finance, daily life and activities, interactions with patient, and mental health. Additionally there was an improvement in all aspects of family functioning such as problem-solving, communication and interpersonal relationship. These positive changes were maintained at 12-month follow-up and were more significant than for the routine care group. The results also indicate similar improvements in all three aspects of patients’ level of functioning in the psychoeducation group while the families in routine care had mild changes in functioning over the 12-month follow-up period.

As mentioned above, most previous family intervention studies have focused on Caucasian populations, and only few studies have been carried out on Chinese or Asian populations, in which great importance is attached to intimate interpersonal relationships and interactions with family members [2]. Thus, this study provides support for the successful application of the existing psychoeducational model of family intervention to a Chinese family-oriented culture. More importantly, the psychoeducation program addressed the specific educational needs of Chinese families, developing a new needs-based educational treatment model for schizophrenia, as suggested by Chien and Wong [7] and Sellwood *et al*. [18]. The results highlight the importance of a family needs assessment, the encouragement of peer support between participants, and adequate staff training as well as on-going supervision for the running of the program, which became the integral parts of the psycho-education program that was used in this study.

Consistent with the findings of this study, research indicates that participation in an education and support group program for family caregivers of people with chronic physical and mental illness is associated with improvements in the psychosocial adjustments of family members [24], better acceptance of the illness and coping with their caregiving role [16,31], and improvements in patients’ physical and mental conditions [1]. Nearly two decades ago, Medvene and Krauss [50] reported that mutual aid groups for families of the mentally ill could increase initiative and comfort in talking with other participants more freely about their psychological and social problems in caregiving. The participants in this study also employed more frequently interactive help-seeking coping strategies, such as more reading and exploration on the problems encountered in caring for the patient and discussion of ways of coping with these caregiving situations. Studies of educational and cognitive-behavioral family interventions for patients with schizophrenia have reported benefits in the areas of decreased burden and increased mastery over the illness [17,54]. However, the effect of mutual support on these families is less frequently discussed. The results of this study on psychosocial functions of the supportive group participants and their patients with schizophrenia are encouraging, and further empirical study using this model of care is recommended in other clinical settings and diverse patient groups.

The lengths of patients’ hospitalizations in the psychoeducation group reduced significantly only over the nine-month intervention period. However, it did not differ between the two groups over the follow-up periods. This finding is consistent with previous randomized controlled trials of family intervention in schizophrenia; family programs involving group approach might show non-significant positive effect on duration and rates of patient readmission when compared both with standard care and with other single modalities [17,32]. Linszen *et al*. [19] have suggested that single-family intervention usually provides support individualized psychological support for family members in terms of: (i) information on the mental illness, (ii) patient management and coping with the illness and its symptoms, (iii) how to identify and solve the specific health problems and (iv) the needs of an individual family accurately. I would argue that more emphasis and time spent on individual family coping with stresses relating to patient management should be incorporated in a family group program to accrue significant benefit for patients’ re-hospitalization [55].

There were also no differences between the two groups in utilization of family support services, which did not change over time. Despite moderate levels of burden and general functioning, the Chinese families in this study reported low levels of formal support service utilization when compared with their Western counterparts [1,21]. This may be explained by the reluctance of Chinese people to reveal family matters and their condition in front of others whom they are not familiar with. Many Chinese families are reluctant to seek professional help, even though they are mostly in need of it [56]. In studies of the effects of mutual support group intervention for Chinese families with a relative with mental illness, Chien *et al*. [2] and Pearson and Ning [16] reported that, over one year or more, a mutual group is effective in offering practical advice and experience and providing information on appropriate community services to meet family needs. Such studies have reported enhanced perceived social support, help-seeking behavior and self-efficacy in caregiving [1,16,22].

In this study, the control group under routine psychiatric care had shown little improvements in most of the families’ and patients’ psychosocial health conditions. These results may be explained by the facts that the routine mental health services in Hong Kong are not provided in a systematic and comprehensive manner according to the families’ needs. Most of the mental health services in the outpatient clinics are run in an ad hoc basis, in which no case managers or nurses are responsible for planning and monitoring the family-centered care and each family requesting any type of service will be referred by the responsible health professional. However, the mental health nurses who provided the routine care to these families were specialized in psychiatric rehabilitation. They demonstrate high level of competency in organizing individual and group therapies for outpatients with severe mental illness and their families, as indicated from the departmental records. As suggested by Anderson and Adams [20] and Chien *et al*. [2], family-centered care for mentally ill clients has not been well-established in clinical practice due to manpower and time constraints, and indeed a sad lack of policies in support of the innovative interventions designed by the health professionals.

Other factors may have contributed to the effects of family psychoeducation and support shown in the study. Studies have reported that contacts and interactions between group participants may affect their emotional support and practical help, which is extended to post-intervention period [23]. Moreover, Maton [57] suggests the importance of understanding the helpful environment within an educational and supportive group for family members of a mentally ill client. Chien *et al*. [22] suggested the need for research on such interactive processes in a family support group context, in which social support and integration are assumed to be shaped by a complex and interrelated network of factors that span multiple psychological as well as social domains and levels of analysis. An exploration of the group processes, in terms of group integrity and development, level of involvement, and internal mutual help and support mechanisms, is essential to better understand the therapeutic components of the psychoeducation and supportive group program.

### Strengths and Limitations of the Study

Despite the random selection of subjects, most of the families in this study were those who were volunteers and highly motivated to participate in the intervention (i.e. very low reported dropout rates in the two groups), with education level of secondary school or above and satisfactory monthly income. This sample may not be representative of those seeking and receiving mental health treatment. In addition, the family caregivers in the psychoeducation group program might provide socially desirable responses on the outcome measures over the follow-up period because they may well be eager to show appreciation and positive feedback to the health professionals and the research team who have offered additional family support. Therefore, caution and future research is needed when interpreting the ‘positive’ findings in this study.

All the subjects were chosen from psychiatric outpatient clinics and had no more than five years of schizophrenia. The content and duration of the program were standardized and no booster sessions were offered over the follow-up period. However, professional input or self-administration of group members has been found to serve as a booster, and seen as important in maintaining the effects of the group intervention on the participants, as suggested by Buchkremer *et al*. [17] and Pearson and Ning [16].

The findings of this study only provided support for the hypotheses that there would be significant statistical differences between the intervention and control groups in terms of families’ burden and functioning as well as patients’ level of functioning over a period of one-year follow-up. Longer term effects of this group program (e.g., at least two years, as planned by the researcher) on improving their health conditions of the families and their relatives with schizophrenia should be examined to further confirm the evidence regarding the effectiveness, importance and usefulness of community-based family intervention in the mental health services.

Finally, the psychoeducation group was led by the research nurse who had expertise in mental health care and group work. If the group had been organized and led by the practicing psychiatric nurses with whom they were familiar, participants might have joined in more readily [20]. If this innovative intervention is integrated into the routine clinical practice and community rehabilitation services, it will enhance the family-centered care in schizophrenia.

Strengths in the design of the study include: (a) randomized treatment allocation with intention-to-treat analysis; (b) clinic staff and an outcome assessor who were blind to the families’ allocation of groups; (c) use of a standardized treatment protocol and guideline for the treatment group and a consistent monitoring system of the group intervention;; and (d) examination of multiple family and patient health-related outcomes. In addition, the study also carefully evaluated the group program, used a systematic approach of longitudinal data collection.

## CONCLUSIONS

While there is a growing body of evidence on the efficacy of psychosocial interventions for schizophrenia, family intervention has indicated clear positive effects on the outcomes of patients’ relapse and readmission. However, it is incumbent upon health care professionals to gather strong empirical evidence about the relative impact of different models of family intervention on psychosocial health outcomes of patients and their family caregivers. The psychoeducation and mutual support group program examined in this study indicated positive effects on family burden and functioning as well as patients’ levels of daily functioning when compared with the standard mental health care. These findings certainly warrant further empirical investigation of this group intervention model, preferably with family caregivers from different socio-economic and cultural backgrounds in Chinese and Asian populations, and in patient groups with chronic schizophrenia or co-morbidity of other mental disorders. Further longitudinal study using mixed methods, consisting of both controlled trial with longer period of follow-up (e.g., more than two-years) and qualitative research approaches, is recommended to explore the long-term effects of the group program not only on improving family caregivers’ health condition but also in schizophrenic clients’ rehabilitation.

## Figures and Tables

**Table 1:** Content Outline of Psychoeducation & Mutual Support Group Program

Session	Goals	Content	Responsible Persons
1 (Family caregivers only)	To orientate to the program and to establish trusting relationship between participants and instructors	Orientation to the program and introduction of group leaders and members to one another Negotiation of goals and roles and responsibilities; Ensuring confidentiality An overview of the topics and their relevance to group members	Research psychiatric nurse
2 (Family caregivers and patients)	To understand schizophrenia, its symptoms and short-term and long-term effects to patients and families	Presentation of a video of one family caring for a person with schizophrenia, with descriptions of symptoms and illness behavior of patient Discussion of the importance of knowledgeable involvement by family members to patient and the whole family. Initial discussion of the mental illness and its effects to family	Research psychiatric nurse
3 (Family caregivers, family members and patients)	To understand the theoretical concepts, etiology and the course of schizophrenia	An overview of theories of schizophrenia from a bio-psychosocial perspective Discussion of the psychiatric conceptualization of schizophrenia, a review of etiology, symptoms, diagnosis, and treatment	Research nurse and one psychiatrist
4-5 (family caregivers only)	To recall and share about illness symptoms and their effects on family life	Information sharing about symptoms and illness behavior; discussion about their effects to family lives Sharing of intense emotions toward patient and suggestion on how to deal with negative emotions to patient An overview of treatments and rehabilitation programs	Research nurse and researcher
6 (family caregivers and patients)	To realize the effects of medications and its compliance	Explanation of positive and negative effects of medications for schizophrenia Explanation of the importance of drug compliance and maintenance Discussion of the specific problems related to the side-effects	Research nurse and one psychiatrist
7-8 (Family caregivers only)	To openly share and more understand about individual concerns and cultural issues	Discussion about Chinese culture of family and mental illness Sharing of intense emotions and feelings about patient care provision and family interactions More information sharing about schizophrenia and its related illness behavior Discuss about the ways to deal with negative emotions to patient	Research nurse
9-10 (family caregivers only)	To improve the family environment and social support	Introduction of the role of environmental stress as a risk factor for acute exacerbations of schizophrenia Discussion of the significance of family as a source of social support and its role and responsibility within the social environment of patient Presentation of family stress and expressed emotion and improvement of family relationships and emotional environment	Research nurse and researcher
11-13 (family caregivers only)	To manage psychosocial needs for themselves, patient and family	Discussion about each member’s psychosocial needs Information about medications, managing illness, and available mental health services Effective communication skills with patient and seeking social support from others Exploration of home management strategies e.g. finance and budgets, environment and hygiene	Research nurse and a clinical psychologist
14-16 (family caregivers only)	To adopt new roles and challenges and skills of coping and patient management	Sharing of coping skills and mutual support Enhancing problem solving skills by working on some individual patient management situations Conducting behavioral rehearsals of interaction with patient and other family members within group Practicing coping skills learned during the sessions to real family life (in-between group sessions) and evaluate the results	Research nurse
17-18 (family caregivers and patients)	To review the previous learning and to prepare for group termination	A review and summary of the materials covered in previous sessions Preparation and discussion on termination issues e.g. separation anxiety, independent living and use of coping skills learned Evaluation of learning experiences and goals achievement Explanation of post-intervention assessment and follow-up taken in the following months	Nurse researcher

Note. Total intervention period of at least 36 weeks.

**Table 2:** Socio-Demographic Characteristics of Families in Psychoeducation Group and Standard Care (N = 68)

Characteristics	Psychoeducation and Support Group (n = 34) a	Standard Care (n = 34)^a^	z Score or t-Test (df = 66), Two Tailed
**Gender**			z = 1.03, p = 0.23
Female Male	21 (61.8) 12 (38.2)	20 (58.8) 14 (41.2)	
**Age**	42.1 ± 6.1	43.2 ± 7.8	t = 1.03, p = 0.10
20-29	6 (17.6)	7 (20.6)	
30-39	12 (35.3)	11 (32.4)	
40-49	12 (35.3)	13 (38.2)	
50 or above	4 (11.8)	3 ( 8.8)	
**Education level**			z = 1.19, p = 0.10
Primary school or below	10 (29.4)	9 (26.5)	
Secondary school	19 (55.9)	20 (58.8)	
University or graduate school	5 (14.7)	5 (14.7)	
**Relationship with patient**			z = 1.00, p = 0.29
Child	10 (29.4)	11 (32.4)	
Parent	9 (26.5)	9 (26.5)	
Spouse	10 (29.4)	10 (29.4)	
Others (e.g. sibling & grandparent)	6 (17.6)	5 (14.7)	
**Monthly household income (HK$)**^b^	16,500± 1,280	13,300± 1,650	t = 1.21, p = 0.12
5,000 – 10,000 10,001 – 15,000 15,001 – 25,000 25,001 – 35,000	6 (17.6) 13 (38.2) 11 (32.4) 4 (11.8)	7 (20.6) 11 (32.4) 12 (35.3) 4 (11.8)	

Note: An independent sample t-test (two-tailed) or z score was used to compare the socio-demographic variables of families between the three groups.

a denotes frequency (f %) or mean ± standard deviation.

b US$1 = HK$7.8

**Table 3:** Outcome Scores at Time 1, 2 & 3 and MANOVA test (Group x Time) Results

Instrument	Psychoeducation Group (n = 34)						Routine Care (n = 34)						F (1, 66)
	Time 1		Time 2		Time 3		Time 1		Time 2.		Time 3		
	M	(SD)	M	(SD)	M	(SD)	M	(SD)	M	(SD)	M	(SD)	
FBIS (0-50) a	29.81	(7.01)	24.69	(6.10)	23.41	(7.92)	30.26	(8.06)	28.49	(6.01)	26.84	(8.10)	4.32*
Financial burden	9.40	(2.98)	8.53	(1.01)	7.52	(1.12)	9.58	(2.08)	9.23	(1.78)	8.80	(1.51)	4.43*
Family routine	4.06	(0.98)	3.35	(0.80)	3.19	(0.71)	4.10	(0.97)	3.80	(0.98)	3.56	(1.00)	4.32*
Leisure	4.73	(1.01)	3.20	(0.88)	3.01	(0.80)	4.32	(0.62)	4.10	(1.11)	3.94	(1.02)	5.03**
Interaction	5.89	(1.10)	4.70	(0.81)	4.31	(0.91)	5.88	(0.78)	5.61	(1.24)	5.58	(1.01)	4.31*
Physical health	2.24	(0.82)	2.08	(0.80)	1.99	(0.75)	2.38	(0.91)	2.03	(0.72)	2.00	(0.76)	3.10
Mental health	4.12	(1.68)	3.64	(0.90)	3.38	(0.82)	4.00	(0.89)	3.86	(1.10)	3.69	(0.90)	4.26*
FAD (7-28)	17.35	(4.53)	21.08	(5.01)	23.69	(4.03)	18.22	(4.45)	19.50	(5.01)	20.59	(5.10)	4.52*
Problem solving	2.45	(0.67)	2.98	(0.89)	3.25	(0.83)	2.33	(0.68)	2.70	(0.70)	2.94	(0.71)	5.01**
Communication	2.33	(0.78)	2.94	(0.98)	3.09	(0.81)	2.49	(0.77)	2.51	(0.54)	2.85	(0.71)	4.89**
Roles	2.58	(0.65)	2.97	(0.77)	3.28	(0.80)	2.67	(0.61)	2.87	(0.88)	2.98	(0.70)	4.30*
Affective responses	2.32	(0.98)	2.88	(0.86)	3.14	(0.97)	2.61	(0.91)	2.89	(0.67)	2.89	(0.80)	4.12*
Affective involvement	2.61	(1.01)	2.99	(0.90)	3.28	(0.89)	2.80	(0.90)	2.95	(0.69)	3.03	(0.98)	4.01*
Behaviour control	2.68	(0.88)	3.03	(0.90)	3.17	(1.00)	2.69	(0.98)	2.73	(0.70)	2.98	(0.70)	4.05*
General functioning	2.39	(0.99)	2.98	(0.72)	3.48	(0.80)	2.45	(0.69)	2.67	(0.61)	2.87	(0.90)	4.93**
FSSI (1-16)	3.81	(1.10)	3.98	(1.03)	4.39	(1.09)	3.79	(1.20)	3.97	(1.12)	4.28	(1.00)	2.33
SLOF (43-215)	126.8	(16.8)	147.7	(25.8)	163.9	(30.1)	121.2	(17.3)	115.1	(20.9)	120.1	(24.8)	4.68 *
Self maintenance	41.9	(10.8)	48.8	(13.1)	56.8	(19.0)	39.2	(13.8)	36.6	(12.9)	38.8	(12.1)	4.88 **
Social functioning	39.2	(11.8)	46.1	(16.1)	52.3	(15.1)	38.1	(11.5)	34.5	(11.0)	39.7	(16.0)	4.42*
Community living skills	46.4	(10.1)	52.8	(17.8)	54.8	(20.3)	43.9	(12.1)	40.7	(12.0)	43.6	(12.0)	4.30 *
Re-hospitalization^b^	27.6	(8.3)	25.8	(10.1)	27.8	10.3)	28.2	(10.3)	28.0	(12.1)	28.6	(13.2)	2.18

Note:

a Possible range of scores of each scale indicated in parenthesis.

b Length of stay in a psychiatric hospital or in-patient unit at Time 1,2 and 3, in terms of average days of hospitalization.

Time 1 = baseline measurement at the start of intervention; Time 2 = one month after intervention; Time 3 = 12 months after intervention.

FAD = Family Assessment Device; FBIS = Family Burden Interview Schedule; SSQ6 = Six-item Social Support Questionnaire; FSSI = Family Support Service Index; SLOF = Specific Level of Functioning Scale.

* p < .01,

** p < .001.
